# The associations between leukocyte, erythrocyte or platelet, and metabolic syndrome in different genders of Chinese

**DOI:** 10.1097/MD.0000000000005189

**Published:** 2016-11-04

**Authors:** Pingping Zhou, Zhaowei Meng, Ming Liu, Xiaojun Ren, Mei Zhu, Qing He, Qing Zhang, Li Liu, Kun Song, Qiang Jia, Jian Tan, Xue Li, Na Liu, Tianpeng Hu, Arun Upadhyaya

**Affiliations:** a Department of Nuclear Medicine; b Department of Endocrinology and Metabolism; c Department of Health Management, Tianjin Medical University General Hospital, Tianjin, Peoples Republic of China.

**Keywords:** erythrocyte, gender, leukocyte, metabolic syndrome (MS), platelet

## Abstract

Leukocyte, erythrocyte or platelet and metabolic syndrome (MS) are closely correlated, and there exist gender differences. We aimed to explore the associations between the hematological parameters and MS in different genders of Chinese. This cross-sectional study included 32,900 participants (20,733 males, 12,167 females) who were enrolled in a health examination. Clinical data including anthropometric measurements and serum parameters were collected. The associations between hematological parameters and MS of both genders were analyzed separately. Odds ratio (OR) of MS was calculated by binary logistic regression models. All hematological parameters were related to MS. With leukocyte and erythrocyte counts rising, the risks of developing MS increased in both genders, which was more obvious in women. For instance, in model 3, the ORs of MS in leukocyte quartiles in females were from 1.333 to 2.045 (*P* < 0.01), while in males, from 1.238 to 1.675 (*P* < 0.01). Platelet seemed as a protective factor in males. Model 1 and model 3 in quartile 2 demonstrated ORs of 0.922 (*P* < 0.05) and 0.912 (*P* < 0.05). However, platelet acted as risk factor in female. For instance, the ORs were 1.253 (*P* < 0.01), 1.461 (*P* < 0.01), and 1.322 (*P* < 0.01) in platelet quartile 4 of all 3 models in female. Gender has influences on the associations between leukocyte, erythrocyte or platelet, and MS. In both genders, higher levels of leukocyte and erythrocyte increased risks of MS. For men, platelet was a protective factor, but for women, platelet seemed as a risk factor.

## 1 Introduction

Metabolic syndrome (MS) is characterized by a constellation of interrelated metabolic disorder including abdominal obesity, hypertension, hypertriglyceridemia, hyperglycemia and decreased high-density lipoprotein (HDL).^[1]^ MS was first defined in 1998 by the World Health Organization.^[2]^ In 2001, the National Cholesterol Education Program Adult Treatment Panel III proposed alternative clinical criteria for defining the MS.^[3]^ The definition for Chinese people was proposed by the Chinese Diabetes Society in 2004.^[4]^ In 2005, the International Diabetes Federation updated Adult Treatment Panel III definition to meet fasting glucose (FG) standard, which was set by the American Diabetes Association and to tailor WC cut-points to specific ethnicity.^[5]^ And in 2009, a consensus criterion was reached by a joint statement from the International Diabetes Federation and American Heart Association and National Heart, Lung and Blood Institute.^[6]^ MS was diagnosed over 3 of 5 factors as following: increased waist circumference (WC) (indicating central obesity), elevated triglycerides (TG), reduced HDL, elevated blood pressure, and elevated FG. MS shows strong association with increased risk of cardiovascular disease (CVD) and predicts the CVD morbidity and mortality.^[7]^ It is also reported as a risk factor for type 2 diabetes mellitus (DM).^[8]^ MS seems to be an inflammatory state and the link between inflammation and insulin resistance plays an important role in a cluster complex of such disorders.^[9]^

The relationship between hematological parameters and MS remains controversial, and has not been discussed extensively. The major hematological parameters include leukocyte, white blood cell (WBC); erythrocyte, red blood cell (RBC); and platelet (PLT), and thrombocyte. For leukocyte, Wu et al^[10]^ found WBC was negatively correlated with HDL and positively with body mass index (BMI) in boys, yet no significance was found in girls. Hsieh et al^[11]^ reported higher level of WBC correlated with significantly higher BMI in both genders, and lower HDL in male subjects only. Kim et al^[12]^ stated that WBC was elevated in male MS subjects yet no association between WBC and MS in female MS subjects. Pei et al^[13]^ found WBC was significantly higher in the group with MS than without MS in both genders. In the case of erythrocyte, some reports demonstrated that in both men and women, RBC was positively associated with MS,^[14]^ while other studies found no such association in both genders.^[12]^ As far as thrombocyte is concerned, PLT counts were found to rise with increasing numbers of MS components in women, yet no similar trends were observed for men.^[15,16]^ However, in another study, higher PLT count was associated with increased prevalence and risk of MS in both genders.^[17]^

The aforementioned inconsistencies raise the need for further research. This study was to illuminate the relationships between hematological parameters (WBC, RBC, and PLT) and MS and to investigate differences in both genders.

## 2 Methods

### 2.1 Design

In this cross-sectional study, we investigated 32,900 participants (20,733 males, 12,167 females) who were enrolled in the general health examination at the Tianjin Medical University General Hospital during the period from 2007 to 2013. Information on medical history, lifestyle, alcoholic intake, and smoking was obtained during interviews. All participants were ostensibly healthy as they reported. To avoid the influence of confounding factors, the exclusion criteria were determined as the followings: subjects with disease history of hematology, liver, kidney, gastrointestine, or oncology; subjects with any diseases or taking any medicine that might affect hematological parameters or metabolism. Written consents were obtained, and the study was approved by the institutional review board and ethic committee of Tianjin Medical University General Hospital. The protocol has been reported in details in our previous publications.^[1,18–21]^

### 2.2 Measurements

Routine physical examinations and fasting blood sample tests were performed when participants visited the hospital. Height, weight, and WC were measured. And BMI was calculated according to the equation: weight (kilograms) divided by the square of height (meters).^[2]^ Blood pressure was measured by using a standard mercury sphygmomanometer after a seated rest for at least 5 minutes. FG, total cholesterol (TC), TG, HDL, low-density lipoprotein (LDL), alanine aminotransferase (ALT), aspartate amino transferase (AST), total bilirubin (TBIL), blood urea nitrogen (BUN), serum uric acid (SUA), and creatinine (Cr) were determined enzymatically by an autoanalyzer (Hitachi Model 7600 analyzer, Hitachi, Tokyo, Japan). WBC, RBC, and PLT were measured on a hemocytometer analyzer (Sysmex Corporation, Kobe, Japan). The blood cells of the participants were all measured by the same method and equipment.

The followings were the laboratory reference ranges for the parameters: FG: 3.6 to 5.8 mmol/L; TC: 3.59 to 5.18 mmol/L; TG: 0.57 to 1.70 mmol/L; LDL: 1.33 to 3.37 mmol/L; HDL: 0.8 to 2.2 mmol/L; ALT: 5 to 40 U/L; AST: 4 to 40 U/L; TBIL: 3.4 to 20 μmol/L; BUN: 1.7 to 8.3 mmol/L; SUA: 140 to 414 μmol/L; Cr: 44 to 115 μmol/L; WBC: 4.0 to 10.0 × 10^9^/L; RBC: 3.5 to 5.5 × 10^12^/L; and PLT: 100 to 300 × 10^9^/L.

### 2.3 Definition

Diagnosis of MS required at least three of the followings: central obesity, WC more than 90 cm in men and more than 80 cm in women; hypertriglyceridemia, TG more than 1.7 mmol/L; low HDL cholesterol, LDL of 1.03 mmol/L or less in males or 1.29 mmol/L or less in females; hypertension, systolic blood pressure (SBP) of 130 mmHg or more, or diastolic blood pressure (DBP) of 85 mmHg or more; and hyperglycemia, FG 5.6 mmol/L or more.^[1,6,18]^

WBC, RBC, and PLT were classified in 2 ways. The first grouping method was done according to medical reference ranges. WBC was divided into 3 subgroups. WBC of 4.0 × 10^9^/L or less was considered as leukopenia, WBC more than 10.0 × 10^9^/L as leukocytosis, and 4.0 × 10^9^/L less than WBC of 10.0 × 10^9^/L or less as normal. Likewise, in accordance with the lower and upper cutoff values of RBC and PLT; abnormalities were termed as erythropenia, erythrocytosis, thrombopenia, and thrombocytosis, respectively. The second grouping method was based on quartiles of the measurements.

### 2.4 Statistical analysis

The analysis was accomplished by using the Statistical Product and Service Solutions (SPSS version 17.0, Chicago, IL). All data were presented as mean ± standard deviation. Independent sample *t* test was used to analyze differences of indices between groups. Multiple comparisons among subgroups were analyzed by the least significant difference test. Chi-square test was used to compare intergroup prevalence differences. Pearson bivariate correlation was made among variables. The binary logistic regression models were used to calculate the odds ratio (OR) for MS with 95% confidence interval. *P* < 0.05 was regarded as significant.

## 3 Result

### 3.1 Characteristics of the participants in different genders

Table 1 revealed that all parameters had significant differences between opposite gender (*P* < 0.01). Males were younger than females. BMI, WC, SBP, DBP, FG, TG, ALT, AST, TBIL, BUN, Cr, and SUA in males were higher than in females. However, TC, HDL, and LDL in males were lower than in females. WBC and RBC in males were higher than in females, yet PLT was lower in males than in females.

**Table 1:**
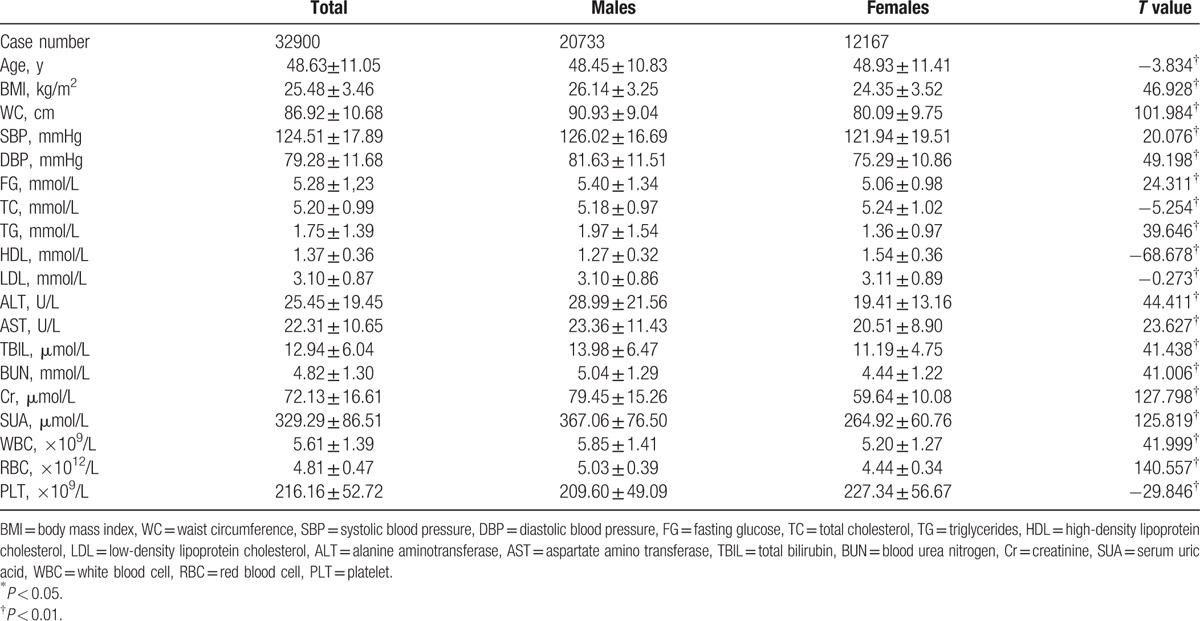
Population characteristics based on different genders.

### 3.2 Prevalence of MS in different genders

Of the 32,900 participants, 32.47% (10,684/32,900) had MS. The prevalence rates of MS in males and females were 37.67% (7811/20,733 cases) and 23.6% (2873/12,167 cases), respectively. It was significantly higher in males than in females, with a chi-square value of 363.387 (*P* < 0.01).

**Figure 1:**
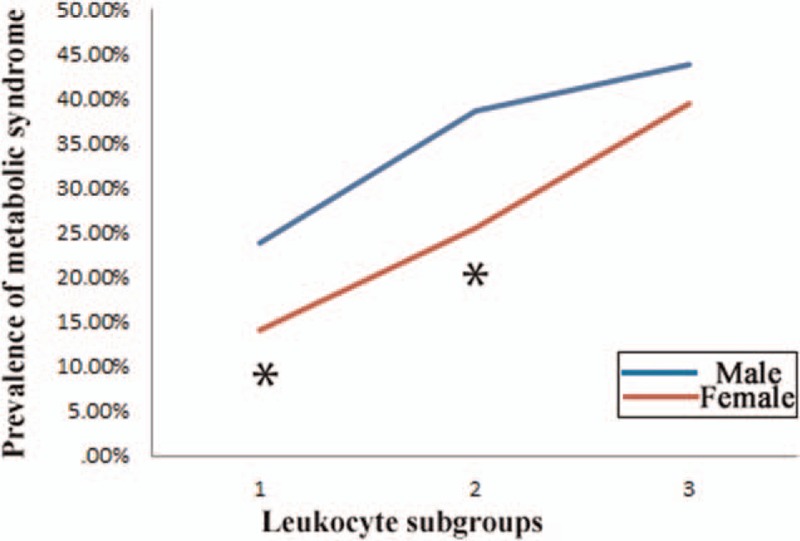
Prevalence of metabolic syndrome in different leukocyte subgroups. Subgroups 1 to 3 referred to the followings: leukocyte of 4.0 × 10^9^/L or less, 4.0 to 10.0 × 10^9^/L, and more than 10.0 × 10^9^/L. ∗ shows significant difference between genders with *P* < 0.01.

According to leukocyte subgroups, except for leukocytosis subgroup, males had significantly higher MS prevalence than females (*P* < 0.01). The prevalence of MS increased as WBC counts increased, which was more prominent in females than in males (for females, chi-square value = 130.640, *P* < 0.01; for males, chi-square value = 119.292, *P* < 0.01) (Fig. 1).

**Figure 2:**
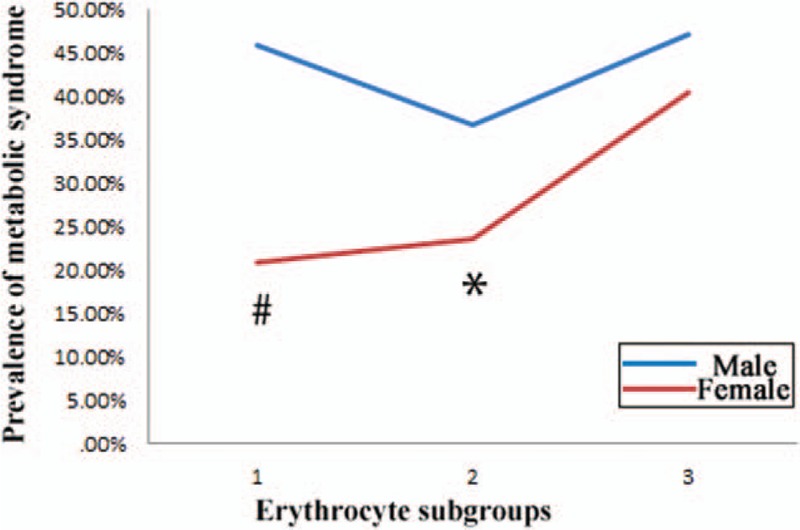
Prevalence of metabolic syndrome in different erythrocyte subgroups. Subgroups 1 to 3 referred to the followings: erythrocyte of 3.5 × 10^12^/L or less, 3.5 to 5.5 × 10^12^/L, and more than 5.5 × 10^12^/L. # shows significant difference between genders with *P* < 0.05. ∗ shows significant difference between genders with *P* < 0.01.

The prevalence of MS according to erythrocyte subgroups revealed different patterns (Fig. 2). Men had significantly higher MS prevalence than women in erythropenia subgroup (*P* = 0.033) and normal RBC subgroup (*P* < 0.01). Prevalence of MS showed an increasing tendency in females, the significantly sharp increase of MS prevalence started from the normal RBC subgroup to erythrocytosis subgroup (chi-square value = 6.809, *P* = 0.033). MS prevalence showed a zigzag pattern in different RBC subgroups in males (chi-square value = 87.916, *P* < 0.01).

**Figure 3:**
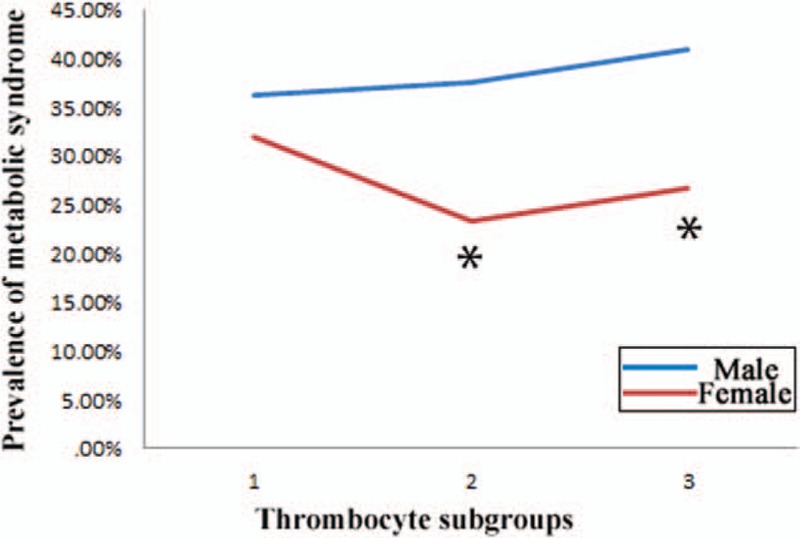
Prevalence of metabolic syndrome in different thrombocyte subgroups. Subgroups 1 to 3 referred to the followings: thrombocyte of 100 × 10^9^/L or less, 100 to 300 × 10^9^/L, and more than 300 × 10^9^/L. ∗ shows significant difference between genders with *P* < 0.01.

As for thrombocyte subgroups, there were significant differences on the prevalence of MS (Fig. 3). The prevalence of MS in males were significantly higher than in females except for the thrombopenia subgroup (*P* < 0.01). The prevalence of MS changed none-significantly in the subgroups (chi-square value = 4.004, *P* = 0.135). However, in females, there was an obvious decreasing trend (chi-square value = 9.628, *P* < 0.01).

### 3.3 Correlations of key variables in different genders

WBC showed significantly positive correlations with BMI, WC, SBP, DBP, FG, TC, TG, LDL, ALT, BUN, and SUA, yet significantly negative relationships with age, HDL, and TBIL in both genders (Table 2).

**Table 2:**
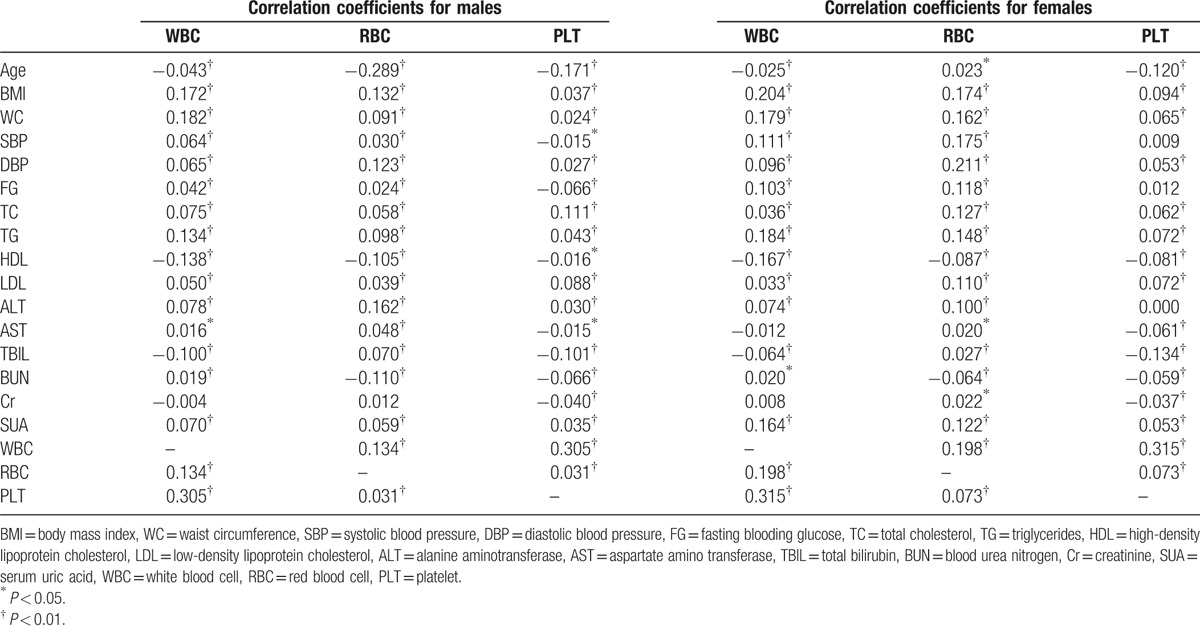
Pearson bivariate correlations among key variables based on different genders.

It was revealed that RBC was positively correlated with BMI, WC, SBP, DBP, FG, TC, TG, LDL, ALT, AST, TBIL, and SUA, yet negatively correlated with HDL and BUN in both genders. Age showed negative correlation with RBC in males, yet positive correlation in females.

In both genders, PLT and BMI, WC, DBP, TC, TG, LDL, and SUA showed positive correlations, while PLT and age, HDL, AST, TBIL, BUN, and Cr showed negative correlations.

### 3.4 Risks of developing MS in different genders

Binary logistic regression models were utilized to calculate the risks of developing MS in different blood cell quartiles (Table 3). WBC, RBC, and PLT quartiles were designated as categorical variables, with the lowest quartile as the reference. Model 1 has no covariate, model 2 included age and BMI as covariates and model 3 included age, BMI, ALT, AST, TBIL, BUN, Cr, and SUA as covariates. For leukocyte, significantly increased risk of developing MS was demonstrated from quartile 2 to 4 for both genders. In general, females had higher ORs than males. And for the same quartile, ORs decreased as covariates increased in both sexes. For erythrocyte, very similar results were demonstrated just like leukocyte. The ORs increased as RBC quartiles rose in both genders. Females had higher ORs than males. And ORs decreased as covariates increased in both sexes. For thrombocyte, our logistic regression models demonstrated some protective effects against MS in males, yet detrimental effects in females. In model 1 and model 3, quartile 2 PLT displayed protective effects for men since ORs were 0.922 (*P* < 0.05) and 0.912 (*P* < 0.05), respectively. Here, protective effects meant lower possibility of developing MS in higher PLT quartiles. For women, higher PLT levels would increase the risk of MS. For instance, ORs of quartile 4 in the models were 1.253 (*P* < 0.01), 1.461 (*P* < 0.01), and 1.322 (*P* < 0.01), respectively.

**Table 3:**
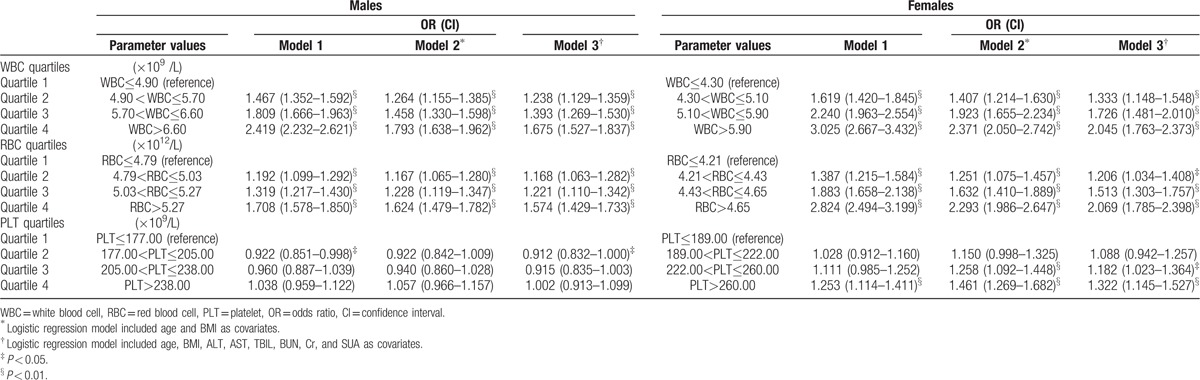
The likelihood of MS in different genders.

## 4 Discussion

The prevalence of MS was increasing not only in well-developed countries but also in developing countries. Multiple studies are necessary to investigate its risk factors. With the significant progress of science and the speedy development of economy, lifestyle of Chinese people has changed obviously. A more calorific diet, a rise of processed food, and a lack of exercise all result in metabolic abnormality, such as obesity, hypertension, and dyslipidemia, which can decrease the quality of life. People with MS are more likely to have heart disease and DM than those who do not have. Type 2 DM and CVDs morbidity are the explicit adverse outcomes of the MS and it is also related to elevated risk for CVDs mortality.^[5,7,8]^

This study showed that WBC count was closely related to MS, the risks of developing MS increased in both genders as the WBC count elevated. Similar performance was done in several previous reports.^[22,23]^ Among women, the risk of MS increased across successive quartiles of WBC counts.^[22]^ The baseline levels of WBC count were significantly higher among MS cases than those without in apparently healthy Korean adults.^[23]^ The mechanisms explaining the association between WBC and MS are not fully elucidated, but several possibilities have been expounded. Insulin resistance, abdominal obesity and inflammation have been mentioned as the major underling factors of MS. Inflammation may be a core mechanism of MS,^[9]^ and it is a link between obesity and insulin resistance.^[24]^ Adipose tissue macrophages are increased in obesity and associated with low grade inflammation.^[25]^ IL-6 and TNF-α secreted by the macrophages are the possible pathogenesis of insulin resistance.^[26]^ Meanwhile, insulin resistance itself increases WBC.^[27]^ These cytokines also increase WBC count,^[26]^ which is a marker of inflammation. This study also demonstrated that the risk of developing MS in WBC quartiles was higher in female than that in male. Although this mechanism also remains unclear, there is an assumption that estrogen might play a role.^[12]^ MS increases risk for CVD. Before menopause, women have lower rates of coronary heart disease than males.^[28]^ Estrogen decreased LDL cholesterol concentration and increased HDL cholesterol concentration.^[29]^ After menopause, however, the loss of endogenous estradiol production could increase the above risks.^[30]^ Men are found to possess higher MS prevalence than women due to lack of the protection of female sex hormone.^[31]^ Nevertheless, female sex hormone has been demonstrated to be positively associated with WBC count, while male sex hormone showed negative association.^[32]^ Taken the information together, female sex hormone seems to be a crucial factor here. The positive influence of female sex hormone to WBC might overcome its protective effect to MS, resulting in the phenomenon of more emphasized risk of rising WBC for MS in females. This may elucidate gender disparity in this study.

Our results also suggested that RBC was significantly correlated with MS. A previous report^[33]^ was in accordance with the present findings, yet another report suggested no relationship existed between MS and RBC.^[12]^ The mechanism between RBC and MS is not clearly explained; however, insulin resistance might also be the key link. Insulin stimulated RBC proliferation, in turn, elevated RBC mass induced insulin resistance.^[34]^ But, why there is a gender difference between RBC and MS? Sex hormone is also hypothesized to play a role. For instance, patients in androgen replacement therapy have a decreased change in RBC.^[35]^ Another report stated that erythrocyte count rose during testosterone treatment.^[36]^ There was a deduction that low-total testosterone levels were positively correlated with MS and type 2 DM, when compared with those who were in higher total testosterone levels.^[37]^ More researches are essential to expose the mechanism between RBC and MS.

In our investigation, gender difference was found in PLT subgroups as well. PLT was identified as a protective factor in male, yet a risk factor in female. Our results were in line with several previous studies. Jesri et al^[38]^ reported that PLT was positively related with the number of MS risk factors. Higher PLT count in women had higher risk of MS development, yet no similar result was observed in male.^[15,16,39]^ Although the reason for the discrepancy by gender in the association of PLT counts with MS remains poorly understood, some explanatory biological mechanisms may be offered. These different roles in male and female could be explained partially by the following possibilities. First, MS is characterized by increased adiposity tissue, which can secrete a variety of adipokines and cytokines such as leptin, adiponectin, interleukin 6, and tumor necrosis factor-α. These proinflammatory cytokines could lead to chronic low-grade inflammation and increase PLT counts. Women generally have a higher fat percentage than men with the same BMI index. Hence, PLT counts may be inherently higher in women. So it can be deduced that PLT is positively correlated with MS in women.^[16,40,41]^ Second, men are found to have higher MS prevalence than women due to lack of the protection of female sex hormone.^[31]^ Female sex hormone is generally believed to decrease the risk of MS. Nagata et al^[42]^ suggested that autocrine estradiol secretion could increase PLT production by triggering pro-PLT formation. This study showed that PLT was positively related with MS developing in women. The role of PLT in female was due to the dominance of female sex hormone on PLT. We consider that the enhancing effect of female sex hormone to PLT might overcome its protective role for MS, resulting in gender disparity in this study. Third, as far as testosterone is concerned, it was reported that administration of testosterone replacement therapy could result in PLT increase ^[43–45]^. But in our study, we did not identify such an endangering effect of PLT on MS in male. So we figure that testosterone might not play as important role as female sex hormone. More researches need to be carried out for confirmation.

The present study has several limitations. First, it was a cross-sectional study, so a causal relationship cannot be pinpointed. Further prospective studies are necessary to explain the causality question. Second, sex hormones were not measured in this investigation due to budget shortage, so the real effects of sex hormones need to be confirmed in the future. Third, cytokines (like interleukin 6 and tumor necrosis factor α) were not measured in this study because of budget shortage as well. Forth, insulin resistance was not gauged, which shall be in our nest stage investigations. Finally, detailed food recall and some other consumed drugs, which could influence hematological parameters or metabolism, should be recorded in specific details for risk stratification in further research.

In conclusion, higher levels of WBC, RBC, and PLT are potential risks for developing MS in females. However, higher levels of WBC and RBC are risks in males, while higher PLT is a protective factor. Special attention should be paid when measuring these easily available hematologic parameters. Higher levels of these easily measured hematologic parameters may play important roles in MS.

## Acknowledgments

This study was supported by the National Key Clinical Specialty Project (awarded to the Departments of Nuclear Medicine and Radiology).

This study was supported by Tianjin Medical University General Hospital New Century Excellent Talent Program; Young and Middle-aged Innovative Talent Training Program from Tianjin Education Committee; and Talent Fostering Program (the 131 Project) from Tianjin Education Committee, Tianjin Human Resources and Social Security Bureau (awarded to ZM).

This study was supported by China National Natural Science Foundation grant 81571709, Key Project of Tianjin Science and Technology Committee Foundation grant 16JCZDJC34300 (awarded to ZM).

This study was also supported by Tianjin Science and Technology Committee Foundation grants 12ZCZDSY20400 and 13ZCZDSY20200 (awarded to QZ and KS).

The funders gave financial support, but did not design the investigation.
